# Supplementary material for: Characteristics of Pathogenic Escherichia coli Associated with Diarrhea in Children under Five Years in Northwestern Ethiopia
Source: Trop Med Infect Dis. 2024 Mar 21;9(3):65. doi: 10.3390/tropicalmed9030065 (PMC10975463; doi:10.3390/tropicalmed9030065)
Supplement: Supplementary file 1 [file tropicalmed-09-00065-s001.zip › tropicalmed-2884192-supplementary.pdf]

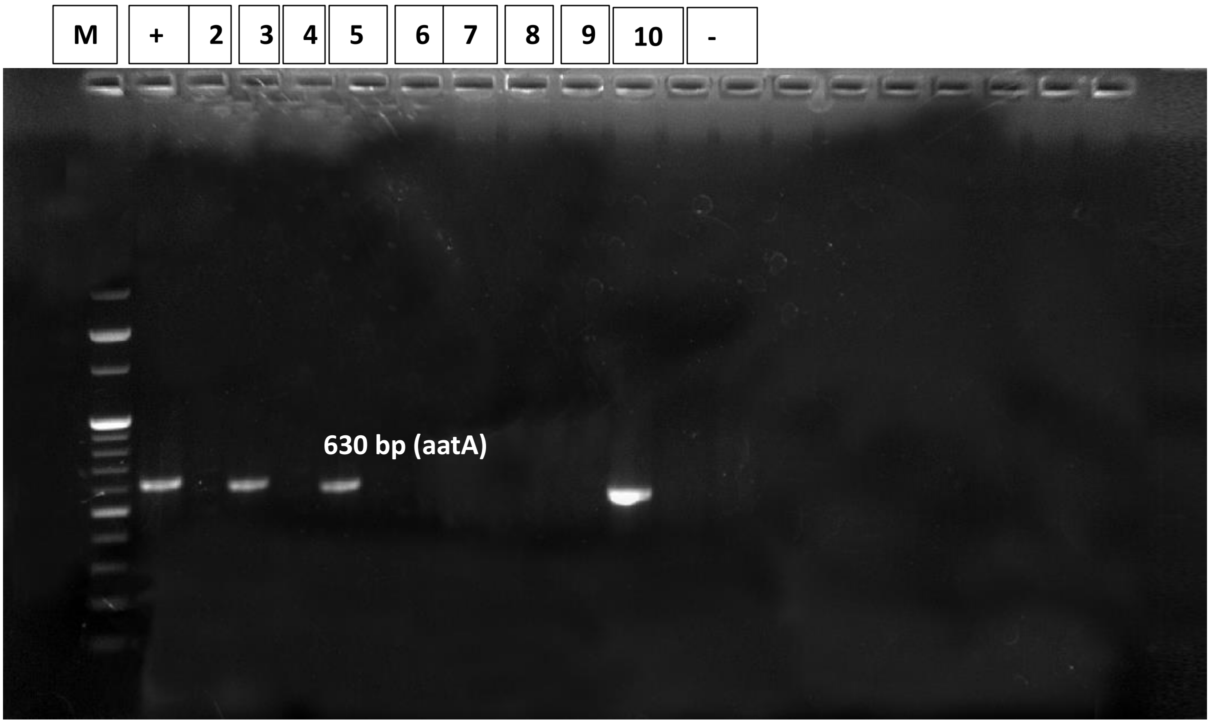
M
+
2
3
4
5
6
7
8
9
10
-
630 bp (aatA)

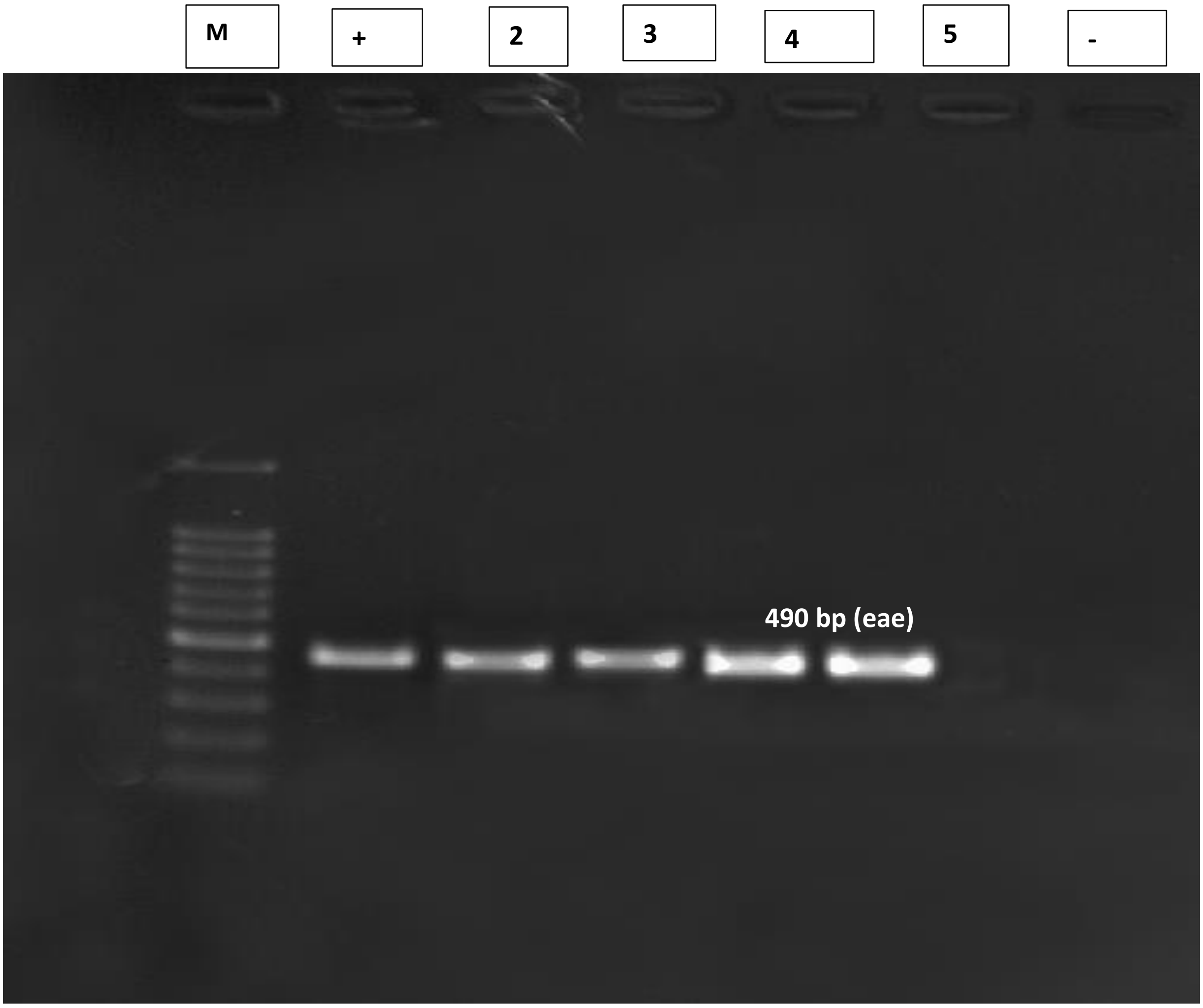
M
+
2
3
4
5
-
490 bp (eae)

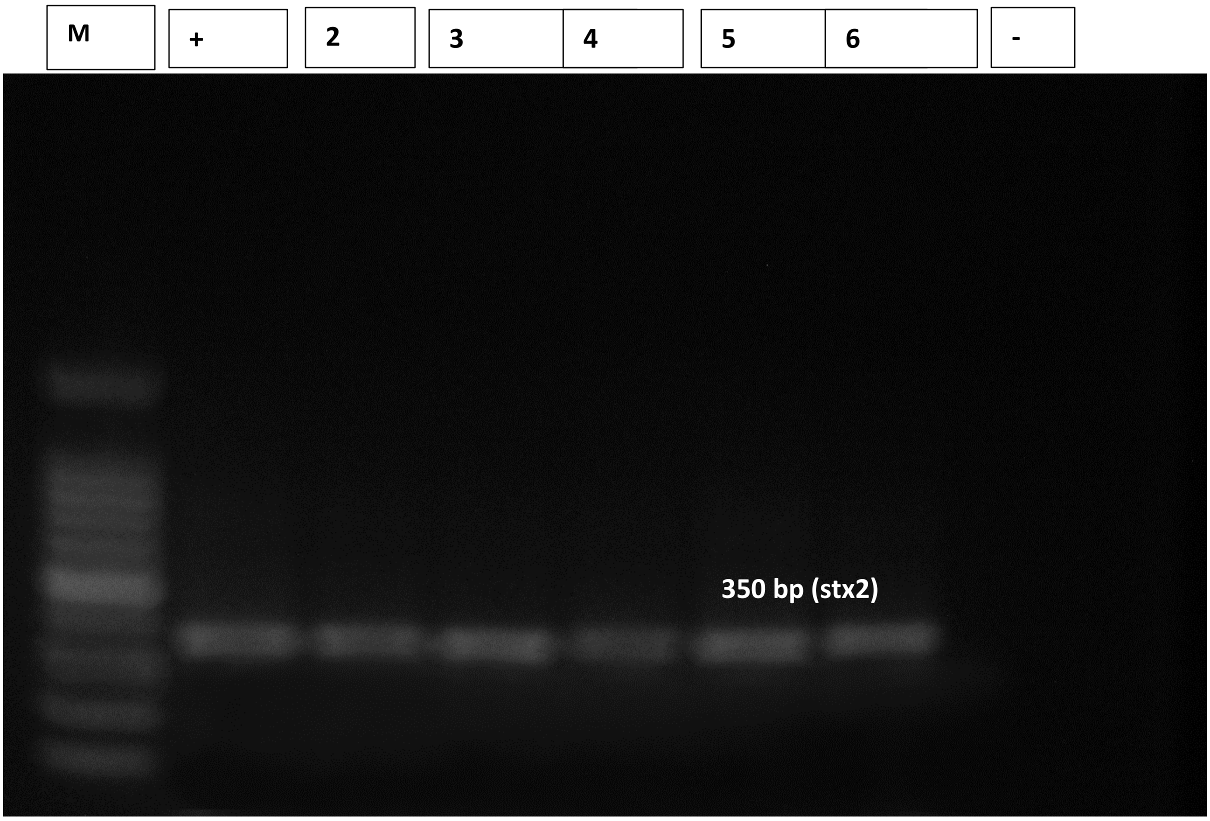
M
+
2
3
4
5
6
-
350 bp (stx2)

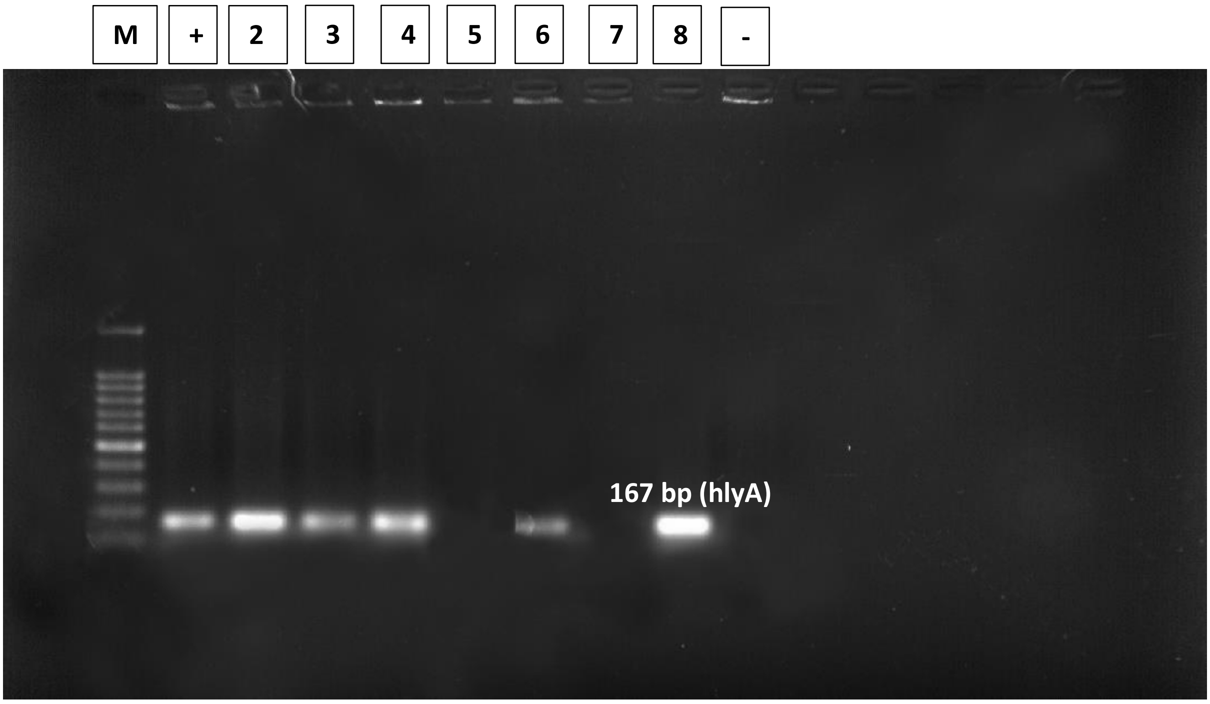
M
+
2
3
4
5
6
7
8
-
167 bp (hlyA)

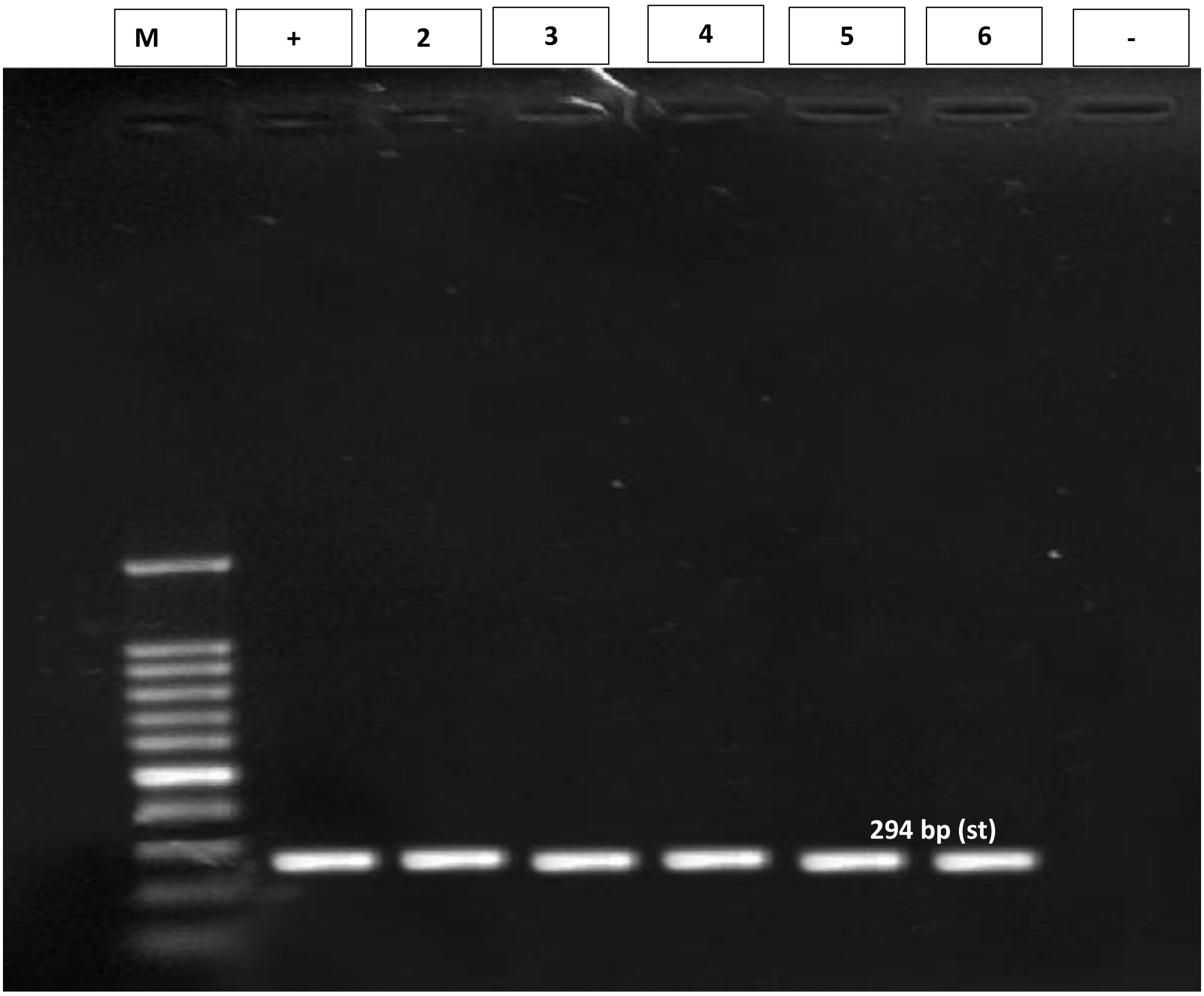
M
+
2
3
4
5
6
-
294 bp (st)

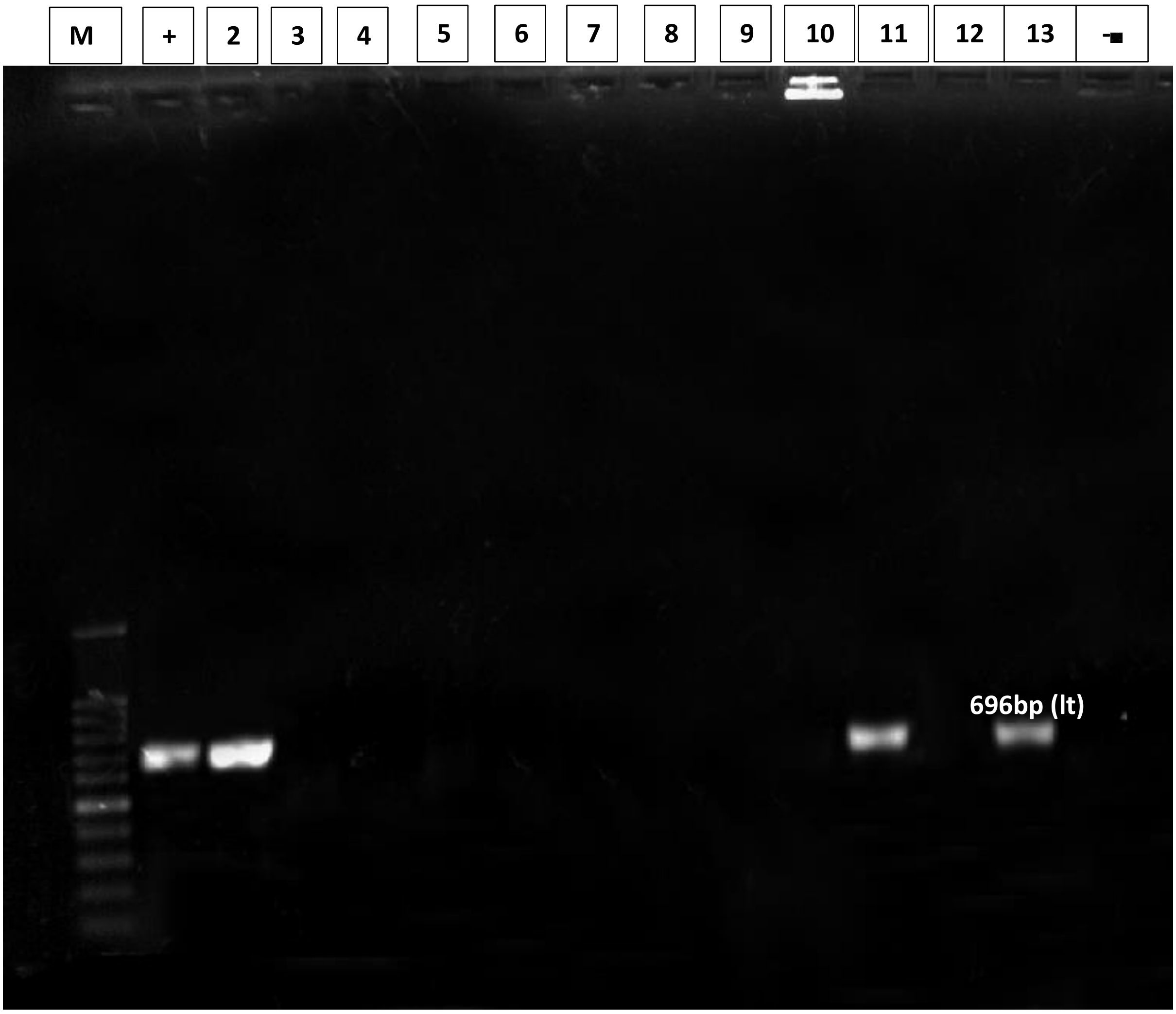

Figure S1. Representative Agarose gel electrophoresis raw images of PCR amplified products. M: marker size (100bp), Lane 1(+): Positive control; Lane 2-13: PCR product of isolates; Lane (-): Negative control
